# Short-term sequence evolution and vertical inheritance of the *Naegleria *twin-ribozyme group I intron

**DOI:** 10.1186/1471-2148-6-39

**Published:** 2006-05-02

**Authors:** Odd-Gunnar Wikmark, Christer Einvik, Johan F De Jonckheere, Steinar D Johansen

**Affiliations:** 1 Department of Molecular Biotechnology, RNA Research Group, Institute of Medical Biology, University of Tromsø, N-9037 Tromsø, Norway; 2 Department of Pediatrics, University Hospital of North Norway, N-9038 Tromsø, Norway; 3 Protozoology Laboratory, Scientific Institute Public Health, B1050 Brussels, Belgium; 4 Department of Fisheries and Natural Sciences, Bodø University College, N-8049 Bodø, Norway

## Abstract

**Background:**

Ribosomal DNA of several species of the free-living *Naegleria *amoeba harbors an optional group I intron within the nuclear small subunit ribosomal RNA gene. The intron (Nae.S516) has a complex organization of two ribozyme domains (NaGIR1 and NaGIR2) and a homing endonuclease gene (NaHEG). NaGIR2 is responsible for intron excision, exon ligation, and full-length intron RNA circularization, reactions typical for nuclear group I intron ribozymes. NaGIR1, however, is essential for NaHEG expression by generating the 5' end of the homing endonuclease messenger RNA. Interestingly, this unusual class of ribozyme adds a lariat-cap at the mRNA.

**Results:**

To elucidate the evolutionary history of the Nae.S516 twin-ribozyme introns we have analyzed 13 natural variants present in distinct *Naegleria *isolates. Structural variabilities were noted within both the ribozyme domains and provide strong comparative support to the intron secondary structure. One of the introns, present in *N. martinezi *NG872, contains hallmarks of a degenerated NaHEG. Phylogenetic analyses performed on separate data sets representing NaGIR1, NaGIR2, NaHEG, and ITS1-5.8S-ITS2 ribosomal DNA are consistent with an overall vertical inheritance pattern of the intron within the *Naegleria *genus.

**Conclusion:**

The Nae.S516 twin-ribozyme intron was gained early in the *Naegleria *evolution with subsequent vertical inheritance. The intron was lost in the majority of isolates (70%), leaving a widespread but scattered distribution pattern. Why the apparent asexual *Naegleria *amoebae harbors active intron homing endonucleases, dependent on sexual reproduction for its function, remains a puzzle.

## Background

*Naegleria *is a common genus of soil and freshwater free-living amoeba of the vahlkampfiid family [1]. *Naegleria *apparently lack a sexual reproduction cycle since meiosis never has been observed or proven experimentally. Subsequently, a number of genetically-defined variants have been isolated in nature and proposed as distinct species [1,2]. A typical *Naegleria *amoeba cell contains a distinct nucleus with a large and predominant nucleolus containing as much as 3000–5000 copies of an approximately 14-kb ribosomal DNA (rDNA) plasmid [3,4]. Each rDNA molecule carries a single transcription unit for the ribosomal RNA (rRNA) genes. Some *Naegleria *isolates have been reported to contain group I intron insertions at conserved sequence sites, both within the small subunit (SSU) and large subunit (LSU) rRNA genes [5]. Introns have been noted at position 516 in SSU rDNA (i.e. a position that is homologous to corresponding position in the *E. coli *rRNA gene) and at positions 1921, 1926, 1949, and 2563 in LSU rDNA [5-11].

Group I introns are autocatalytic genetic elements carrying a ribozyme domain responsible for the intron self-splicing reaction, and occasionally a homing endonuclease gene (HEG) encoding an endonuclease protein directly involved in intron mobility at the DNA level [12,13]. A group I splicing ribozyme possess a well-defined three-dimensional structure organized into three functional domains (catalytic domain, folding domain, and substrate domain) by approximately ten paired RNA segments named P1–P10 [14,15]. The most common and best characterized of the *Naegleria *rDNA introns is Nae.S516. Group I introns at position 516 in SSU rDNA are relatively common among eukaryotic microorganisms with more than 250 cases reported so far [16,17], and with both lateral and vertical inheritance patterns compared to that of host rDNA. A widespread distribution and structural diversity among the S516 group I introns have been noted, including several complex introns carrying HEGs [16,18].

A typical *Naegleria *S516 intron has a twin-ribozyme organization and represents the most complex class of all group I introns known [19]. Nae.S516 consists of a small group I-like mRNA capping ribozyme (NaGIR1) and a HEG domain, both inserted into the P6b segment of a regular group IC1 splicing ribozyme (NaGIR2). Expression and functional aspects of the *Naegleria *S516 intron have been reported. The splicing ribozyme (NaGIR2) is responsible for the autocatalytic activity that generates intron excision and exon ligation, as well as full-length intron RNA circle formations [7]. The ability to form full-length intron RNA circles is a general property of nuclear group I introns and could be important in RNA mobility at the RNA level, or even as an intermediate in the expression of the homing endonuclease [11,20,21]. The *Naegleria *HEG (NaHEG) encodes a 245 amino acid protein that belongs to the His-Cys box family of homing endonucleases [22,23]. The intron endonuclease recognizes and binds to a 19-bp DNA sequence flanking the S516 rDNA site and cleaves the DNA generating a five-nucleotide 3' staggered end [24,25]. In general, group I intron endonucleases promote intron homing at the DNA-level by generating a double-stranded break in the intron-less target DNA, followed by invasion of the donor intron-containing allele and DNA repair using the intron-containing allele as template [21]. However, sexual mating is the biological framework for nuclear group I intron homing [26,27] and it is unclear why the apparently asexual *Naegleria *contains and maintains homing introns.

The expression of the NaHEG is dependent on a functional NaGIR1 ribozyme, which defines the 5' end of the homing endonuclease mRNA by internal processing and modification of the excised Nae.S516 intron [7,28,29]. Primer extension analyses of both cellular RNA from *Naegleria *and *in vitro *transcribed intron RNA [7,28] are consistent with the formation of a tiny lariat cap structure between nucleotide 1 and 3 of the messenger, as recently reported in the related DiGIR1 ribozyme [30]. Thus, the NaGIR1 capping ribozyme represents a novel class of ribozymes possessing a new catalytic function, which is reflected in its unique RNA architecture [29,31].

The complex and unique structural organization of the *Naegleria *twin-ribozyme intron makes it interesting to investigate the evolutionary origin of the different intron domains, as well as the inheritance pattern within the *Naegleria *genus. Here we report several new intron variants and have performed sequence and phylogenetic analyses providing new insight into fundamental questions such as intron structure, intron-host biology, and the origin and evolution of intron HEG and ribozyme domains. The *Naegleria *twin-ribozyme intron serves as an attractive model system in the characterization of evolutionary processes behind a recently gained, but vertically inherited, selfish genetic element.

## Results and discussion

### Widespread but sporadic distribution of Nae.S516 introns within the *Naegleria *genus

**Figure 1:**
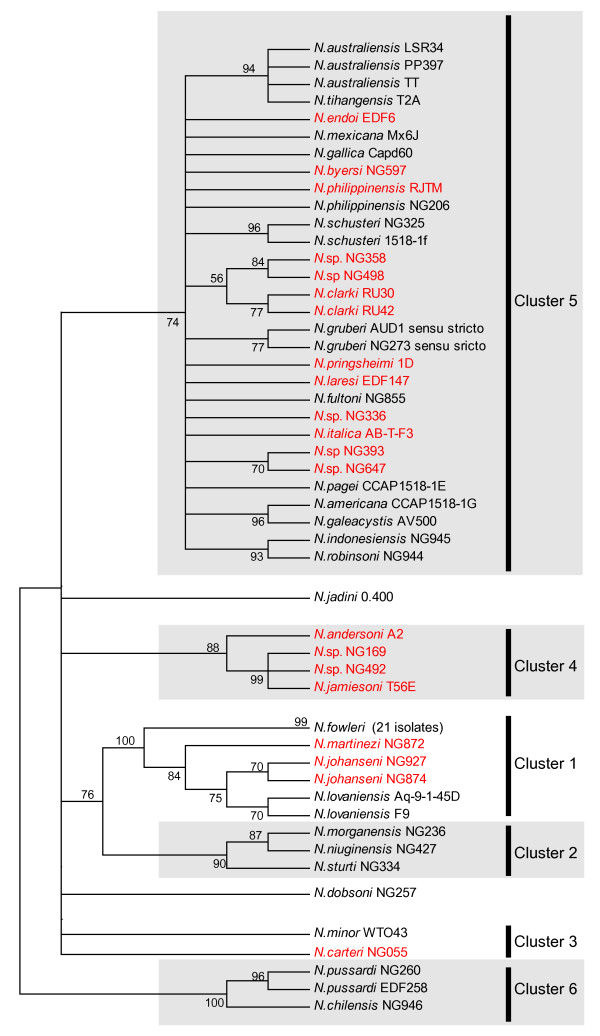
**Distribution of the Nae.S516 introns among *Naegleria *isolates**. NJ phylogenetic tree of the ITS-rDNA based on 287 nucleotide positions and 70 *Naegleria *isolates. NJ bootstrap values above 50% are shown at the branches. The six clusters of *Naegleria *isolates (Cluster 1–6) are indicated according to [1,2]. *Naegleria *isolates known to contain (21 isolates) or lack (49 isolates) the Nae.S516 intron are shown in red or black, respectively. All introns are approximately 1,3 kp in size (corresponding to a twin-ribozyme organization), except *N. byersi *NG597 which is 375 bp (only NaGIR2). The *N. fowleri *branch in Cluster 1 represents 21 distinct isolates.

Sequence analysis of ITS-rDNA from 70 natural isolates of *Naegleria *(Table 1) was performed to gain insight into the genetic relationships among strains and species. A phylogenetic tree based on the NJ method is presented in Figure 1, and corroborates previous finding of six main clusters of *Naegleria *isolates [1,2]. Most clusters appear monophyletic with high bootstrap and Bayesian supports, and Cluster 5 is the most prominent with 30 annotated *Naegleria *isolates (Figure 1). A closer inspection of the SSU rRNA identified intron sequences inserted at position S516 in 21 of 70 strains analyzed. All introns, except one [8], belong to the highly complex twin-ribozyme group I intron family [7,19]. Group I introns at position 516 in SSU rDNA are relatively common among eukaryotic microorganisms [19]. Interestingly, the *Naegleria *516 introns (Nae.S516; for intron nomenclature see [32]), show a widespread but scattered distribution among the *Naegleria *isolates (Figure 1). All clusters, except the early diverging Cluster 6 [2], harbor isolates that carry the group I intron. Furthermore, no linkages could be noted between the presence of the S516 intron and environmental factors such as optimal growth temperature, pathogenesis, habitats, and geographical origin of *Naegleria *isolates.

### Structural features and sequence variability of intron domains

**Figure 2:**
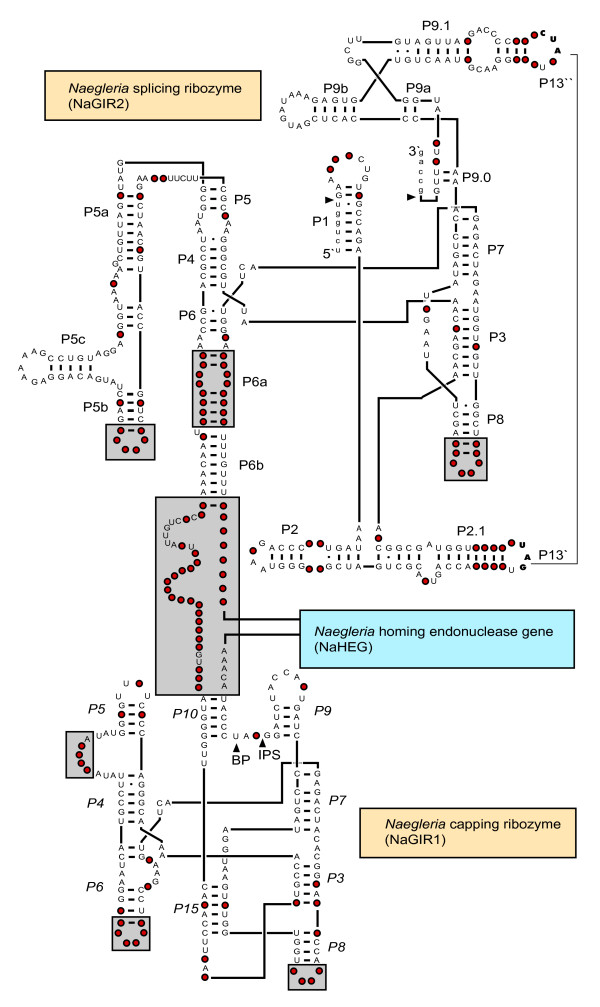
**Consensus structure diagram of the Nae.S516 twin-ribozyme intron**. The structure diagram is based on the 13 completely sequenced Nae.S516 introns from distinct natural isolates (see Table 1) and is folded according to previously reported models [7,19] with some modifications. Invariant nucleotide residues are presented as uppercase letters. Red filled circles represents a variable position in one or more intron sequences, and regions with size and structural variations are boxed. Nae.S516 contains the two distinct ribozymes NaGIR1 and NaGIR2, and the homing endonuclease gene NaHEG. IPS, internal processing site; BP, branch point nucleotide (U).

Fourteen of the 21 introns were selected for more detail structural characterizations (see Table 1). All introns, but one (*N. byersi *NG597), possess the twin-ribozyme group I intron organization previously reported [7,16], and an updated RNA consensus structure diagram is shown in Figure 2. The intron consists of three functional domains, identified as distinct intron structures. The autocatalytic *Naegleria *splicing ribozyme (NaGIR2) is responsible for intron excision and exon ligation during precursor rRNA processing in the *Naegleria *nucleolus, as well as for the generation of circular intron RNAs [7,28]. NaGIR2 represents a typical group IC1 intron with clear structural resemblance to the well-studied *Tetrahymena *ribozyme [14,15]. The consensus structure (Figure 2) is strongly supported by compensatory base substitutions among the *Naegleria *introns. The most prominent differences between the *Naegleria *and *Tetrahymena *ribozymes are the lack of a P9.2 segment in *Naegleria*, the presence of an optional tetra-loop in L5b, and a large sequence insertion (approximately 950 nt) in P6b harboring the homing endonuclease gene (NaHEG) and the capping ribozyme NaGIR1 (Figure 2).

NaGIR1 is a group I-like ribozyme with an evolved biological role in intron NaHEG expression [29,30], likely to generate a lariat cap-structure at the 5' end of the *Naegleria *homing endonuclease messenger [30]. The three-dimensional architecture of NaGIR1 is related to that of bacterial tRNA group I intron ribozymes [18,29,33], but with a unique catalytic core organization that contains the novel pseudoknot segment P15 [7,29,31,34]. As seen from Figure 2, most of the core nucleotides are highly conserved among the various *Naegleria *capping ribozymes. Variable regions are almost exclusively located in the terminal loops of P6 and P8, the internal loop junction J5/4, and sequences flanking NaGIR1 and NaHEG.

**Figure 3:**
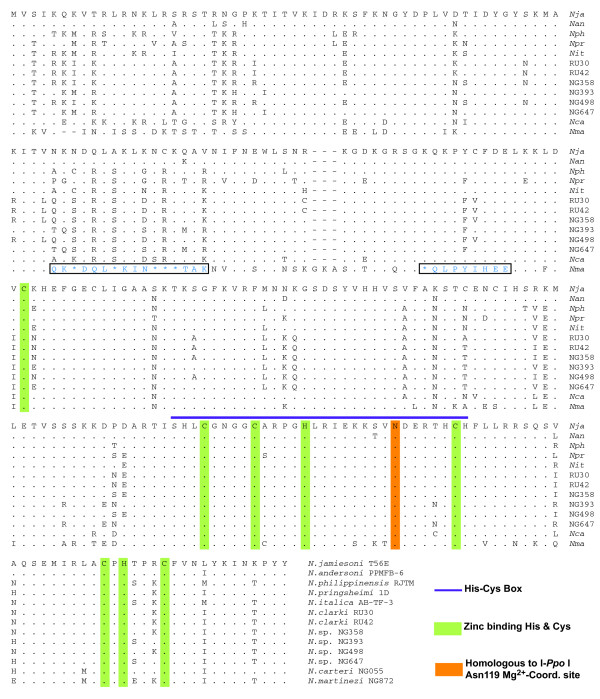
**Alignment of *Naegleria *homing endonuclease sequences**. Identical residues compared to the *N. jamiesoni *T56E endonuclease sequence are shown by dots and deletions by dashes. Functional important residues involved in zinc binding and catalysis are indicated. Divergent regions within the *N. martinezi *NG872 sequence due to reading-frame shifts (*) are boxed. For structural comparisons to the I-*Ppo*I homing endonuclease, see [23].

The third intron domain consists of the NaHEG which codes for a 245 amino acid (aa) His-Cys endonuclease that recognizes and cleaves the intron lacking allele sequence in rDNA [24,25]. An alignment of the derived amino acid sequence from the studied *Naegleria *introns is presented in Figure 3. The amino acid identities between pairs vary from 81 % to 100 %, with the positive charged N-terminal region (first approximately 80 aa) as the most variable part. The N-terminal region contain arginine and lysine rich sequences that resembles known RNA binding domains [35,36], indicating that the NaHEG encodes a complex protein with both endonuclease and RNA binding functions. All three intron domains at the RNA-level (NaGIR1, NaGIR2, endonuclease mRNA) are possible targets for intron protein RNA binding. Both NaGIR1 and NaGIR2 are known to fold correctly and to be catalytically active *in vitro *without assistance of proteins [7,31,34], suggesting no essential maturase function of the intron encoded protein. However, the intron protein could still be able to bind to it own messenger, which is predicted to be highly structured [7]. This possibility remains to be further experimentally explored. Residues previously noted to be essential for endonuclease active site definition, catalysis, and zinc coordination (Figure 3) [25] are highly conserved. The non-synonymous to synonymous substitution rates (dN/dS) were calculated for the NaHEG sequences and found in all cases to be below one (data not shown), indicating purifying selection. Interestingly, the intron present in *N. martinezi *NG872 isolate contains hallmarks of a degenerated NaHEG, seen as multiple frame shifts and small indels (Figure 3).

### The splicing ribozyme of Nae.S516 intron is vertically inherited in *Naegleria*

**Figure 4:**
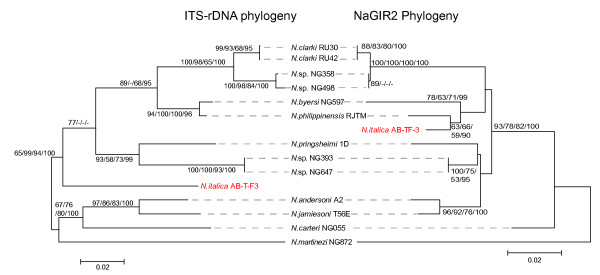
**Phylogeny of NaGIR2 and ITS-rDNA**. The NaGIR2 and ITS-rDNA NJ trees were generated from 356 and 415 nt datasets. Bootstrap values from 2000 replicates and Bayesian posterior probability values, all over 50%, are shown at branches. The values are from, in order, NJ, MP, ML, and BAY analyses. See Materials and Methods for more detailed analytical parameters. ITS-rDNA and NaGIR2 phylogenies are congruent, except for *N. italica *AB-T-F3 (marked in red). See Table 1 for information about sequence accession numbers.

NaGIR2 represents the splicing-ribozyme domain of Nae.S516 and thus corresponds to the sequences present in a prototype group I intron such as the *Tetrahymena *intron. We have previously performed phylogenetic analysis of various nuclear S516 group I introns, including five NaGIR2 variants, and recognized the *Naegleria *introns as a monophyletic clade among the group IC1 introns [16]. Here we extend the analysis to include 14 NaGIR2 variants representing the different isolates of *Naegleria*. Furthermore, we also include a host rDNA analysis based on their corresponding ITS-rDNA sequences. The intron phylogeny was based on 356 sequence positions within NaGIR2, strictly aligned according to the structure diagram in Figure 2. Different methods (NJ, MP, ML, and BAY) were used to build the phylogenetic trees, and all trees were essentially identically in topology. Similarly, the ITS-rDNA phylogeny was based on 415 sequence positions using the same set of tree building methods described above in intron analysis. Figure 4 shows representative NJ trees of both the ITS-rDNA and NaGIR2 phylogenies with overall congruent branching patterns and significant bootstrap and Bayesian supports. The only exception is *N. italica *AB-TF-3 (Figure 4), that could represent a recent horizontal intron transfer. However, the *N. italica *ITS-rDNA branch topology is only poorly supported in NJ analysis (77 %) and without bootstrap and Bayesian supports in the MP, ML, and BAY analyses, respectively. Thus, we infer there are no experimental support of horizontal intron transfer and conclude that the NaGIR2 domain, representing the Nae.S516 intron, is vertically inherited within the *Naegleria *genus.

### The capping ribozyme NaGIR1 and its downstream NaHEG are evolutionary linked

**Figure 5:**
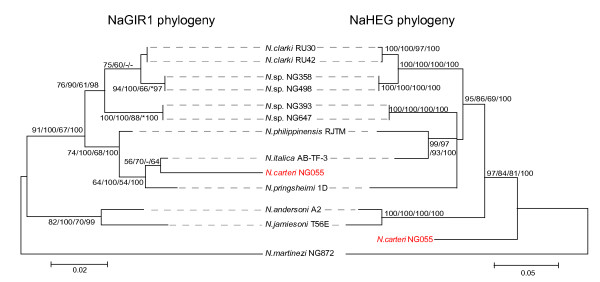
**Phylogeny of NaGIR1 and NaHEG**. The NaGIR1 and NaHEG NJ trees were generated from 230 and 747 nt datasets. Bootstrap values from 2000 replicates and Bayesian posterior probability values, all over 50%, are shown at branches. The values are from NJ, MP, ML, and BAY analyses, respectively. See Materials and Methods for more detailed analytical parameters. NaGIR1 and NaHEG phylogenies are congruent, except for *N. carteri *NG055 (marked in red). See Table 1 for information about sequence accession numbers.

Divergent evolutionary histories of group I splicing ribozymes and their HEGs have been described in several nuclear group I introns [37-39]. The *Naegleria *twin-ribozyme intron is highly unusual among group I introns in that it contains two distinct structural domains (NaGIR1 and NaHEG) inserted into the same peripheral region (P6) of NaGIR2 (see Figure 2). To address the relationships among the variants of NaGIR1 and NaHEG, as well as between the different intron domains (NaGIR1, NaGIR2, and NaHEG) we performed phylogenetic analyses based on the sequence alignments. Figure 5 presents representative NJ trees of NaGIR1 and NaHEG. The phylogenetic trees, built by the MP, ML, and BAY methods, were essentially identical. The NaHEG tree was based on 747 aligned positions and possesses significant bootstrap and Bayesian supports. The total size of the NaGIR1 domain is less than 250 nucleotides in size and thus only 230 positions could be included in the analysis. However, we found the tree topology to be well supported in bootstrap and posterior probability analyses. Interestingly, the NaGIR1, NaHEG, and NaGIR2 (compare Figures 4 and 5) phylogenies were found to have congruent branching topologies consistent with a co-evolutionary pattern of the domains within introns. But there is one clear exception in *N. carteri *NG055 (see Figure 5), suggesting a homologous recombination-like event between natural sequence intron variants.

A *Naegleria *S516 group I intron with only NaHEG or only NaGIR1 insertions has never been observed, suggesting a strong linkage between the domains. Both structural and functional data give further support to a close linkage between the NaGIR1 and NaHEG domains. Jabri and Cech [34] showed that the RNA structure essential for NaGIR1 catalysis includes nucleotide residues within the NaHEG coding region. Functional experiments in yeast conclude that expression of NaHEG, and subsequent endonuclease activity in yeast extracts, is completely dependent on a functional NaGIR1 ribozyme [28]. Thus, the NaGIR1 and NaHEG domains have to be considered as one functional unit within the Nae.S516 intron.

### Gain of a L5b GNRA tetraloop in NaGIR2 during *Naegleria *evolution

One of the best-studied tetraloop receptor interaction is the L5b-P6a tertiary structure in the *Tetrahymena *group I intron ribozyme [40]. Here, the GAAA loop in L5b specifically binds to the 11-nt receptor motif CCUAAG-UAUGG within the helical stem of P6a by docking into the shallow groove. The L5b-P6a interaction in *Tetrahymena *is essential for an efficient folding of the P4–P6 domain, and subsequently the folding and activity of the splicing ribozyme.

**Figure 6:**
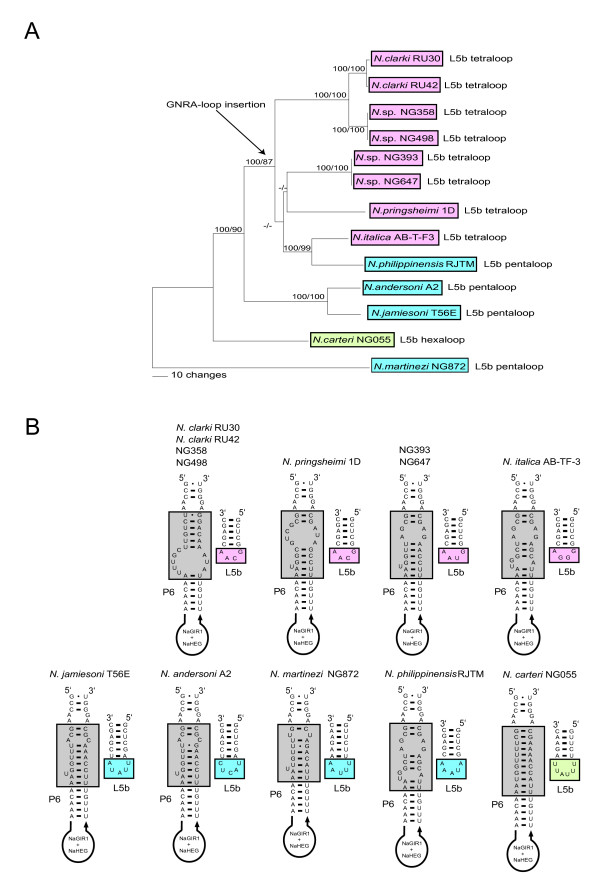
**Structural variations in L5b and P6a of NaGIR2**. A) NJ phylogenetic tree of the 13 complete Nae.S516 introns based on 1370 positions. NJ and MP bootstrap values are indicated at the branches. The presence of L5b tetra- (red), penta- (blue), or hexaloops (green) in L5b in NaGIR2 are indicated. All tetra-loops belong to the GNRA-loop family. B) Structure diagrams of P6a (grey boxes) and L9b representing the 13 twin-ribozyme Nae.S516 introns.

Two of the most variable parts in the *Naegleria *GIR2 splicing ribozyme are L5b and P6a (Figure 2). A closer inspection of the 13 twin-ribozyme intron sequences identify tetra-, penta-, and hexaloops in L5b of 8, 4, and 1 introns, respectively. All tetraloops belong to the GNRA family (N = A, G, C or U; R = A or G) known to specifically interact with receptor sequences. To address the distribution pattern of the L5b tetraloops among the various *Naegleria *intron isolates a phylogenetic analysis based on 1370 positions, representing the complete twin-ribozyme introns, was performed. Essential identical trees were obtained from the NJ and MP methods, and a representative NJ tree is shown in Figure 6A. Interestingly, introns harboring a L5b GNRA tetraloop cluster together with high bootstrap support, suggesting that a L5b tetraloop was gained late the evolution of the *Naegleria *genus. The only exception appears the L5b pentaloop of the *N. philippinensis *RJTM intron (Figure 6A), but this example may represent a secondary loss of a GNRA tetra-loop (e.g. GUAA to AUAAA).

The primary function of GNRA tetraloops is to participate in long-range RNA-RNA interactions by specific binding to a receptor motif. A variety of receptor motifs, ranging from 4 to 12 nt, have been recognized experimentally [41-43]. Figure 6B presents secondary structure diagrams of the various NaGIR2 P6a regions and their corresponding L5b loops. A prominent difference in the P6a structure is noted among introns possessing L5b GNRA tetraloops compared to those with penta- or hexaloops. Introns with GNRA loops contain a less tightly base-paired P6a stem with several proposed exposed residues (see Figure 6B) compared to the P5b penta- or hexaloop containing introns (compare *N. clarki *RU30 and *N. carteri *NG055). We speculate that the exposed residues in P6a could be involved in RNA-RNA interactions as GNRA receptors. However, these sequences do not fit any known consensus motifs, suggesting that new motifs are yet to be experimentally identified.

## Conclusion

Evolutionary aspects on the structural organization of *Naegleria *twin-ribozyme group I introns have been reported previously [19]. Whereas the NaGIR2 splicing ribozyme appears related to other eukaryote rDNA group IC1 intron [16], the NaGIR1 capping ribozyme has recently evolved from a bacterial tRNA group I introns [29]. Here we present phylogenetic evidence of a vertical inheritance pattern of the Nae.S516 intron in *Naegleria *that includes each of the domains NaGIR1, NaGIR2, and NaHEG, and corroborates a previous study based on 5 intron sequences [6]. Based on the reported distribution pattern and phylogeny, we propose the following vertical inheritance scenario for the Nae.S516 evolution. 1) A pre-organized twin-ribozyme group I intron was gained in the rDNA early in evolution of the *Naegleria *genus, but after the Cluster 6 branching (see Figure 1). 2) Once established, the *Naegleria *intron co-evolved along with its host rDNA by maintaining intron activities including intron splicing, endonuclease mRNA capping, and homing endonuclease DNA cleavage. 3) The intron was subsequently lost (see Figure 1) by sporadic deletions in most isolates (70 %). 4) Degradation of the NaHEG is initiated due to loss of biological function, and subsequent selection pressure (e.g. *N. martinezi *NG872), resulting in complete deletion of the NaHEG as well as its regulatory NaGIR1 capping ribozyme (e.g. *N. byersi *NG597) [8]. 5) The remaining introns have to improve and adjust their functions by continuous sequence evolution in order to be maintained within rDNA. Here, a recent gain of a GNRA tetraloop receptor in the P4–P6 domain would facilitate folding of the splicing ribozyme (Figure 6).

What is the biological role of a functional NaHEG in *Naegleria *S516 introns? There are only two reported examples addressing the biological role of nuclear group I intron HEGs in experimental settings. In sexual matings between intron-containing and intron-lacking strains of either the myxomycetes *Physarum polycephalum *or *Didymium iridis*, group I introns were shown to be mobile due to the double-strand-break-repair pathway induced by intron-encoded homing endonucleases [26,27]. In both cases the homing endonucleases were found to cleave the group I intron lacing alleles in a highly sequence specific manner, resulting in unidirectional transfers of introns into the intron-lacking strains. This process is dependent on a sexual reproduction of the host organism, which is apparently absent in *Naegleria*. However, the *Naegleria *intron endonucleases possess hallmarks linked to a function in intron homing. First, sequence comparisons show that the *Naegleria *enzymes belong to the same His-Cys homing endonuclease family as the known homing endonucleases I-*Ppo*I and I-*Dir*I encoded by the mobile *Physarum *and *Didymium *group I introns [10,11,25]. Second, the *Naegleria *endonucleases cleave only intron lacking alleles flanking the intron insertion site at the SSU rRNA gene [24,25]. Finally, artificial expression of the *Naegleria *endonuclease and its intron in yeast generate intron homing intermediates consistent with a homing endonuclease function [28]. Interestingly, *Naegleria *may occasionally perform sexual reproduction in nature since Pernin and co-workers [44,45] reported evidence for genetic exchange in *N. lovaniensis*, including chromosomal recombination. Both haploid and diploid strains of the *N. gruberi *NEG isolate have been described based on both amoeba DNA content and UV-sensitivity [46,47]. Perhaps the observed recombination-like feature of NaGIR1 in *N. carteri *NG055 (see Figure 5) is a result of rare sexual mating. This possibility remains to be experimentally explored.

## Methods

### *Naegleria *strains, DNA amplification, plasmid cloning, and DNA sequencing

The following *Naegleria *isolates were DNA sequenced at ITS-rDNA and Nae.S516 intron regions in this study: *N. clarki *RU30 (ITS-rDNA and Nae.S516); *N. clarki *RU42 (ITS-rDNA and Nae.S516); *N. pringsheimi *1D (ITS-rDNA and Nae.S516); *N. philippinensis *RJTM (ITS-rDNA and Nae.S516); *N. carteri *NG055 (ITS-rDNA and Nae.S516); *Naegleria *sp. NG647 (ITS-rDNA and Nae.S516); *Naegleria *sp. NG358 (ITS-rDNA and Nae.S516); *Naegleria *sp. NG393 (ITS-rDNA and Nae.S516); *Naegleria *sp. NG498 (ITS-rDNA and Nae.S516); *Naegleria *sp. NG169 (ITS-rDNA);*Naegleria *sp. NG336 (ITS-rDNA); *Naegleria *sp. NG491 (ITS-rDNA); *Naegleria *sp. NG492 (ITS-rDNA). The strains without designation are under revision and will be proposed proper species names based on phylogenetic analyses of ITS1-5.8S-ITS2 sequences (De Jonckheere et al. in preparation). A complete list of all 70 *Naegleria *isolates included in this study is presented in Table 1. DNA samples of *Naegleria *strains were prepared as described previously [2], dissolved in water, and applied as template in 50 μl standard PCR reactions. Amplified product of interest were plasmid cloned into the pGEM^®^-T Easy Vector System I (Promega). Individual clones where DNA purified and sequenced with the ABI PRISM BigDyeTerminator Cycle Sequencing Ready Reaction Kit (Perkin-Elmer), running on an ABI Prism 377 system (Perkin-Elmer), or using the sequencing service from MWG Biotech [48]. Two or more individual clones were sequenced from all introns and ITS-rDNA regions analysed. The following oligoprimers were used in Nae.S516 PCR amplifications and DNA sequencing analyses: OP 25 (5'-CTC GAA TTC GCT CTT GGA GCT GGA ATT A-3'), OP 26 (5'-ACG AAG CTT ATT TCT AAG CCT-3'), OP 28 (5'-CAG AGG AGT TTC TTA CCT ATC-3'), OP 131 (5'-AAA CGA ATT CTA TTG ATT AGT AGT-3'), OP 946 (5'-GAA TTG AAA AAG CTT GAT-3'), OP 1200 (5'-AAA CAA ATG CTA TTG ATC A-3'), OP 1201 (5'-GAA CGT CTA GAG ACT ACA CGG-3'), OP 1042 (5'-CGA TTT TCC ATG ATT TGG G-3'), OP 1043 (5'-ATA CCT CAA CAG AGG TCC-3'), OP 1044 (5'-GGA CGT CTA GAG ACT ACA CGG-3'), OP 1045 (5'-TGA TGC ACG TAC GAA TCG GAG C-3'), OP 276 (5'-GGT AAA CAA ATC CCT GTT-3'), OP 823 (5'-TAA CCA TTT TGT ATG GGA-3'). Heteroplasmic rDNA alleles (intron-containing/intron lacing) were not observed. The following oligoprimers were used in ITS-rDNA PCR amplifications and DNA sequencing analyses: OP 918 (5'-AAC CTG CGT AGG GAT CAT TT-3'), OP 919 (5'-TTT CCT CCC CTT ATT AAT AT-3').

### Sequence alignment and phylogenetic analysis

Multiple alignment of sequences were performed by using Megalign (Version 5.06) included in the Lasergene package from DNASTAR, Inc [49], Bioedit (Version 7.0.4.1; [50]), manuel refinements. Phylogenetic analyses and non-synonymous to synonymous substitution rates [51] were conducted using MEGA version 2.1 [52], PAUP* (Version 4.0 Beta) [53], and MrBayes (version 3.1) [54,55]. Trees were built with the methods of neighbor-joining (NJ) using different distance matrixes, maximum parsimony (MP) with the branch and bound search method, as well as Bayesian analyses (BAY) and maximum likelihood (ML) using different evolutionary models. The reliabilities of the tree topologies were evaluated by bootstrapping (NJ, MP, and ML), and posterior probability (BAY).

### ITS and intron sequence analyses

Two different data sets of the internal transcribed rDNA spacer region (ITS-rDNA: ITS1-5.8S-ITS2) were used. In analysis with all the 70 *Naegleria *isolates, only 287 nucleotide positions could unambiguously be aligned due to high sequence variation in ITS2. However, when the analysis was restricted to the 14 intron-containing *Naegleria *isolates we extended the ITS-rDNA region to 415 nucleotide positions. Based on the multiple sequence alignment a NJ tree was constructed with the Kimura-2 evolutionary model of substitution (K2), with pair wise deletion of gaps and bootstrapped with 2000 replications with a cut-off value of 50%. Similarly, intron trees are constructed with NJ-K2 parameters. The robustness of the tree topologies were tested by the NJ-K2 parameter (first value), MP branch and bound search criteria (second value), and ML with the HKY+G model of substitution selected by Modeltest 3.7 [56], all from 1000 replicates. The last values where constructed by running 1000000 generations of Metropolis-coupled Markov chain Monte Carlo, and trees were sampled every 100 generations (average standard deviation of split frequencies below 0.01). A consensus tree was generated from the 75% last trees to find posterior probabilities (Burn-in value = 2500).

## Authors' contributions

OGW did the sequencing of ITS-rDNA, and group I introns in collaboration with CE. OGW performed the phylogenetic analyses. JFD contributed with DNA preparations of *Naegleria *isolates. SDJ directed the research and wrote the paper in collaboration with OGW.
